# Draft Genome Sequences of *Bacillus anthracis* Strains Stored for Several Decades in Japan

**DOI:** 10.1128/genomeA.00633-15

**Published:** 2015-06-18

**Authors:** Akiko Okutani, Makoto Osaki, Daisuke Takamatsu, Yoshihiro Kaku, Satoshi Inoue, Shigeru Morikawa

**Affiliations:** a Department of Veterinary Science, National Institute of Infectious Diseases, Tokyo, Japan; b Bacterial and Parasitic Diseases Research Division, National Institute of Animal Health, National Agriculture and Food Research Organization, Tsukuba, Ibaraki, Japan; c The United Graduate School of Veterinary Sciences, Gifu University, Gifu, Gifu, Japan

## Abstract

We report the draft genome sequences of *Bacillus anthracis* strains Shikan-NIID, 52-40-NIAH, and 44-NIAH stored in Japan and belonging to the A3 cluster.

## GENOME ANNOUNCEMENT

Anthrax caused by *Bacillus anthracis*, a spore-forming bacterium, is one of the most severe zoonoses, which poses serious threats to both public and animal health. The genetic diversity of *B. anthracis* is limited since the bacteria remain as dormant spores in soils for several decades. To elucidate the limited variation between recent and old isolates, we analyzed three strains stored in the National Institute of Animal Health: Shikan-NIID, 52-40-NIAH, and 44-NIAH. Strains Shikan-NIID and 52-40-NIAH were isolated from horses in 1928 in Tokyo, Japan (1), and in 1940 in China (2), respectively. Strain 44-NIAH has no record of its origin.

We first checked that these strains possess both pXO1 and pXO2 plasmids by PCR for *pag* and *cap* genes, respectively, since many *B. anthracis* strains are known to lack one or both plasmids during long-term storage (3). The genomic DNA was extracted from the bacteria cultured overnight in LB broth using the phenol-chloroform method.

The whole-genome sequence of the Shikan-NIID strain was determined by 2 single-molecule real-time (SMRT) cell runs of PacBio RS II P4C2 technology (4) and assembled by the hierarchical genome assembly process (HGAP) (5). The data was assembled to be a total of 5.4 Mbp in three contigs, consisting of chromosome, pXO1, and pXO2. The total length of the mapped region of strain Shikan-NIID was 5,228,065 bp with 5,564 putative coding sequences (CDS) for the chromosome, 181,769 bp with 191 CDS for pXO1, and 94,818 bp with 109 CDS for pXO2.

Libraries for strains 52-40-NIAH and 44-NIAH for Illumina Miseq (Illumina, Inc) analysis by 151-bp paired-end sequencing with Miseq reagent kit v2 (300 cycle) were prepared using a NEBNext ultra DNA library prep kit for Illumina (NEB, Tokyo, Japan) with index primers from NEBNext Multiplex Oligos for Illumina, according to the manufacturer’s instruction.

A total of 3,489,578 reads for strain 52-40-NIAH and 11,579,864 reads for strain 44-NIAH were generated, and the reads that passed the Illumina quality filters were assembled by CLC Genomic Workbench 7.5.1. The assembled contigs were ordered to the Ames “Ancestor” genome (6) as the reference using Mauve 2.3.1 (7). Each ordered genome was annotated using Microbial Genome Annotation Pipeline (MiGAP) (8). Finally, strain 52-40-NIAH sequence data were assembled into 36 contigs with a draft genome length of 5,449,821 bp with 5,813 CDS. Strain 44-NIAH sequence data were assembled into 124 contigs with a draft genome length of 5,375,137 bp with 5,695 CDS.

The whole-genome analysis of the decades-old strains in the present study may contribute to comparative studies of recent *B. anthracis* strains in Japan (9) and other Asian countries (10, 11).

### Nucleotide sequence accession numbers. 

The whole-genome shotgun projects have been deposited in DDBJ/ENA/GenBank, with the accession numbers AP014833 (chromosome), AP014834 (pXO1), and AP014835 (pXO2) for Shikan-NIID, BBWY01000001 to BBWY01000036 for strain 52-40-NIAH, and BBWX01000001 to BBWX01000124 for strain 44-NIAH.
